# Synergistic antibacterial effects of exopolysaccharides/nickel-nanoparticles composites against multidrug-resistant bacteria

**DOI:** 10.1038/s41598-023-48821-y

**Published:** 2023-12-06

**Authors:** Javier A. Garza-Cervantes, Gricelda Mendiola-Garza, Angel León-Buitimea, José Rubén Morones-Ramírez

**Affiliations:** 1 Facultad de Ciencias Químicas, Universidad Autónoma de Nuevo León, UANL, 66455 San Nicolás de los Garza, NL Mexico; 2 Centro de Investigación en Biotecnología y Nanotecnología, Facultad de Ciencias Químicas, Universidad Autónoma de Nuevo León, Parque de Investigación e Innovación Tecnológica, 66629 Apodaca, Nuevo León Mexico

**Keywords:** Antimicrobials, Microbiology, Nanoscience and technology

## Abstract

The need for an alternative treatment to fight infectious diseases caused by antibiotic-resistant bacteria is increasing. A possible way to overcome bacterial resistance to antibiotics is by reintroducing commonly used antibiotics with a sensitizer capable of enhancing their antimicrobial effect in resistant bacteria. Here, we use a composite composed of exopolysaccharide capped-NiO NPs, with antimicrobial effects against antibiotic-resistant Gram-positive and Gram-negative bacteria. It potentiated the antimicrobial effects of four different antibiotics (ampicillin, kanamycin, chloramphenicol, and ciprofloxacin) at lower concentrations than their minimal inhibitory concentrations. We observed that the Ni-composite synergistically enhanced, fourfold, the antibacterial effect of kanamycin and chloramphenicol against multidrug-resistant *Staphylococcus aureus* and *Pseudomonas aeruginosa*, as well as ampicillin against multidrug-resistant *Staphylococcus aureus*, and ciprofloxacin against multidrug-resistant *Pseudomonas aeruginosa* by eightfold. We also found that Ni-composite could not inhibit biofilm synthesis on the tested bacterial strains. Our results demonstrated the possibility of using metal nanoparticles, like NiO, as a sensitizer to overcome bacterial antibiotic resistance.

## Introduction

The golden era of antibiotics allowed pharmaceutical companies to achieve large-scale production of various biologically active compounds, addressing the fight against microbial infections^1^. However, misuse of antibiotics has resulted in the rapid rise of antimicrobial resistance. The ESKAPE pathogen group conformed of *Enterococcus faecium*, *Staphylococcus aureus*, *Klebsiella pneumoniae*, *Acinetobacter baumannii*, *Pseudomonas aeruginosa*, and *Enterobacter* species, have become world-relevant pathogens since they include high resistant bacterial strains^2–4^. The spreading of these antibiotic-resistant strains embodies a critical issue in which international healthcare systems spend millions of dollars annually^5^. An attractive strategy relies on reintroducing previously used antibiotics with different antimicrobial agents, nanomaterials, or sensitizers^6^. The green synthesis of nanomaterials has proven to be a profitable and environmentally friendly alternative, the latter being the main advantage compared to classical synthesis methods (chemical and physical)^7^. Remarkably, synthesizing metallic nanoparticles using bioactive agents, such as plant materials, microorganisms, and various biological wastes, has provided a rapid and cost-effective biosynthetic protocol for synthesizing stable metallic nanoparticles^8,9^. An example is the microbial exopolysaccharides (EPS), which due to their biocompatibility, non-toxicity, and biodegradability, have been used in the green synthesis of various metallic NPs as capping agents^10,11^.

Different methods have been used for synthesizing nickel nanoparticles (NiNPs), such as chemical precipitation, electrodeposition, microemulsion technique, photocatalytic reduction, coprecipitation methods, and microwave irradiation. However, most of these methods have the following disadvantages: complex processes, complex reaction conditions, high temperatures, and long reaction times^12^. Recently, NiNPs have been synthesized using different natural sources (for example, polysaccharides, flavonoids, terpenoids, polyphenols, glycosides, proteins, and vitamins), which act as reducing and coating agents, improving their antimicrobial properties^13,14^. Moreover, the antibacterial activity of biosynthesized nickel oxide nanoparticles has been evaluated in Gram-negative and Gram-positive bacteria, showing promising results^14–17^.

Therefore, in this work, we hypothesized that a biocomposite composed of EPS-capped NiO NPs^11^ (Ni-composite) could enhance (synergistic effect) the antimicrobial effect of four different antibiotics (ampicillin, kanamycin, chloramphenicol, or ciprofloxacin) at lower concentrations than their minimum inhibitory concentration (MIC). Our results demonstrated positive antimicrobial effects of the Ni-composite with ampicillin, kanamycin, and chloramphenicol against a multidrug-resistant *Staphylococcus aureus* (*SaR*) and kanamycin, chloramphenicol, and ciprofloxacin against a multidrug-resistant *Pseudomonas aeruginosa* (*PaR*). Most of these combinations showed a synergistic interaction between Ni-composite and the antibiotic. The antibacterial effects observed by combining different antibiotics with EPS-capped NiO NPs open ups the possibility of using metal nanoparticles synthesized by green methods as an alternative to combat antibiotic-resistant bacterial infections.

## Materials and methods

### Material and microbial strains

Antibiotics ampicillin (AG Scientific Inc.), kanamycin (AG Scientific Inc.), ciprofloxacin (Sigma-Aldrich), and chloramphenicol (Bio Basic, Canada Inc.) stock solutions were prepared following the supplier recommendations. The bacterial strains were *Staphylococcus aureus* ATCC 6538, *Pseudomonas aeruginosa* ATCC 27853, multidrug-resistant *Staphylococcus aureus* (*SaR*; kanamycin, chloramphenicol, ciprofloxacin, and ampicillin-resistant strain), and multidrug-resistant *Pseudomonas aeruginosa* (*PaR*; kanamycin, chloramphenicol, and ciprofloxacin-resistant strain). Multidrug-resistant clinical isolates were kindly provided by the clinical laboratory of Hospital de San Vicente, Monterrey, Nuevo León, México. Strains were grown on Luria Bertani (Difco, USA) broth (LB) adjusted to pH 7 at 37 °C–150 rpm for 16 h to obtain an overnight culture.

### EPS extraction

The microbial EPS (obtained from *Rhodotorula mucilaginosa* UANL-001L) used as a capping agent was produced by incubating 1 mL of an overnight culture (16 h at 30 °C) into 300 mL of Yeast Mold medium (YM) for 96 h at 30 °C and 150 rpm. The supernatant was recovered by centrifuging the culture at 12,000 rpm for 20 min at 4 °C, and then the EPS precipitated by adding 3:1 absolute ethanol:supernatant and storing the mixture at − 20 °C overnight. The EPS was then obtained by centrifugation at 12,000 rpm for 20 min at 4 °C and drying in a SPD2010 SpeedVac concentrator (ThermoFisher Scientific, USA) for 5 h at 5.1 torr with heating at 45 °C for 1 h.

### Synthesis of nanoparticles

Synthesis of Ni nanoparticles in EPS was carried out using nickel sulfate (Productos Químicos Monterrey, México), with ascorbic acid (Jalmek, México) in the presence of microbial EPS extracted from *R. mucilaginosa*, as reported previously^11^. Briefly, 10 mM NiSO_4_, 5 mg/mL of EPS, and 4% w/v ascorbic acid were mixed in an aqueous solution, adjusted to pH 9 with NaOH, and heated for 4 h in a boiling water bath. A blank solution containing K_2_SO_4_ instead of NiSO_4_ was used as a control with the same ascorbic acid and EPS concentrations. Then, the resulting solution was added to a container with three volumes of absolute ethanol and stored at − 20 °C for 1 h, centrifuged at 12,000 rpm for 15 min at 4 °C, and at the end, washed three times with 70% ethanol. The synthesized nanoparticles were dried in a SPD2010 SpeedVac concentrator (ThermoFisher Scientific, USA) for 5 h at 5.1 torr with heating at 45 °C for 1 h.

### Material characterization

UV–Vis spectra were obtained using a Multiskan-GO (Thermo Scientific, USA) in the 200–800 nm range. The Fourier transform-infrared spectroscopy (FTIR) was performed in an IRAfinity-1 (Shimadzu, USA). Morphological and structural characterization was made using transmission electron microscopy (TEM) and selected area electron diffraction. The elements were analyzed using the energy dispersive spectrometry analyzer (EDS) integrated into the FEI-TITAN 80–300 microscope operated at an accelerating voltage of 300 kV. For these techniques, the sample was prepared by depositing and evaporating a drop of the synthesized biocomposite (1 mg/mL), previously dispersed using an ultrasonic cleaner (BRANSONIC, Branson 2510MT), onto lacey carbon-coated copper grids. The diameter of the nanoparticles was measured using ImageJ software (Nation Health Institute).

### Determination of minimum inhibitory concentration

Minimum inhibitory concentration of the different compounds^12^ was determined in 96-well plates (Costar, Corning) based on a modified methodology reported by Andrews^18^ and CLSI^19^ as follows. We added the necessary antibiotic volume from the stock solutions to achieve the needed concentration within a final volume of 200 µL. Next, serial dilutions were made by taking 100 µL to every next well with 100 µL of fresh culture media and discarding 100 µL from the least dilution. This way, the tested concentrations were 8192 to 1 ppm of each antibiotic after adding the resistant or ATCC bacteria inoculum. The ATCC strains were used to compare antibiotic susceptibility against the resistant bacteria.

Similarly, we prepared a stock of 8 mg/mL Ni-composite and EPS alone in LB adjusted to pH 7 with phosphate buffer 0.1 M as the vehicle. We added the necessary volume of the compound in LB to achieve concentrations of 4, 3, 2, and 1 mg/mL as the final concentration once the resistant bacteria inoculum was added.

The inoculation of each test well was made as follows. 100 µL of an overnight culture of each strain was transferred to a tube with 5 mL of fresh media and incubated until it reached a critical optical density (OD_600_ of 0.2 ± 0.02), adjusting with fresh media if necessary and reaching a cellular concentration range 10^7^–10^8^ cells/mL, supported by plate counting observations measured by serial dilution method. From this, a 1:100 dilution was made with fresh media in a 1.5 mL tube, then 100 µL of this dilution was added to each test well to achieve a final concentration of 10^5^ cells/mL, incubated at 37 °C–150 rpm. After 20 h of incubation at these conditions, the ODs of control and treated inoculums were measured, and the MIC was the value at which no significant growth was observed (OD_600_ < 0.05). All tests, and their respective sterility and growth controls, were performed in replicates of three.

### Checkerboard assays of Ni-composite/antibiotic combinations (Ni-CACs)

Combinatory inhibition assays were performed through checkerboard assays^20^ in 96-well plates to observe the synergistic effect of Ni-composite and the four different antibiotics (ampicillin, kanamycin, chloramphenicol, and ciprofloxacin). We used MIC fractions of each component, 0, 0.125, 0.25, and 0.5 MIC, to create the combinatorial treatments for each strain. The MIC fractions of Ni-composite were placed along the abscissa axis of the plate, and the antibiotic MIC fractions along the ordinate axis of the plate using concentrated solutions so that when added to the culture, the needed volume of inoculum MICs fractions were reached. The inoculation of each test well was made as follows. 100 µL of an overnight culture of each strain (*SaR* or *PaR*) were transferred to a tube with 5 mL of fresh media and incubated until it reached a critical optical density (OD_600_ of 0.2 ± 0.02), adjusting with fresh media, if necessary, to reach a cellular concentration range between 10^7^ and 10^8^ cells/mL, supported by plate counting observations measured by serial dilution method. From this, a 1:20 dilution was made with fresh media in a 1.5 mL tube, then 20 µL of this dilution was added to each test well to achieve a final concentration of 10^5^ cells/mL, incubated at 37 °C–150 rpm. After 20 h of incubation at these conditions, the ODs of control and treated inoculums were measured, and their respective values were recorded. Each Ni-CAC, and their respective sterility and growth control samples, were performed in replicates of three (n = 3).

The degree of the synergy of Ni-composite and the four different antibiotics was analyzed using the Bliss independence model described by Hegreness et al.^21^ which states that the interaction can be considered synergistic when the combined effect of the antimicrobial agents is greater than the predicted effect of its components. The value of S describes the interaction between combinatorial treatments as follows: S > 0 Synergistic; S = 0 Additive; S < 0 Antagonistic.

### Inhibition of biofilm production by Ni-CACs

The inhibition of biofilm production assays was performed using the combinations tested for their antimicrobial activity and analyzed by a crystal violet staining^22^. The combination preparation was formulated and inoculated as delineated in the preceding sections. However, it was incubated at 37 °C for a duration of 40 h under non-agitated conditions. Additionally, a controlled humidity environment was maintained to preclude the desiccation of the wells. Non-treated bacteria and bacterial growth control were included on the same plate. After incubation, each well and control supernatant was washed three times with ultrapure water. The plates were heat dried, and each combination well was stained with 240 µL of crystal violet 0.1% for 20 min under static conditions. Then, the dye was removed from each plate, washed three times with ultrapure water, and heat dried. Biofilms were de-stained using an ethanol 99% solution for 30 min under static conditions. From this, 100 µL of each combination's ethanol/crystal violet solution and control well were transferred to another plate, and the optical density was measured at 590 nm. All tests, as well as the untreated controls, were performed in triplicates.

### Data analysis

All collected data were analyzed by ANOVA and Fisher's least significant difference (LSD) tests using Microsoft Excel 2016.

## Results

**Figure 1:**
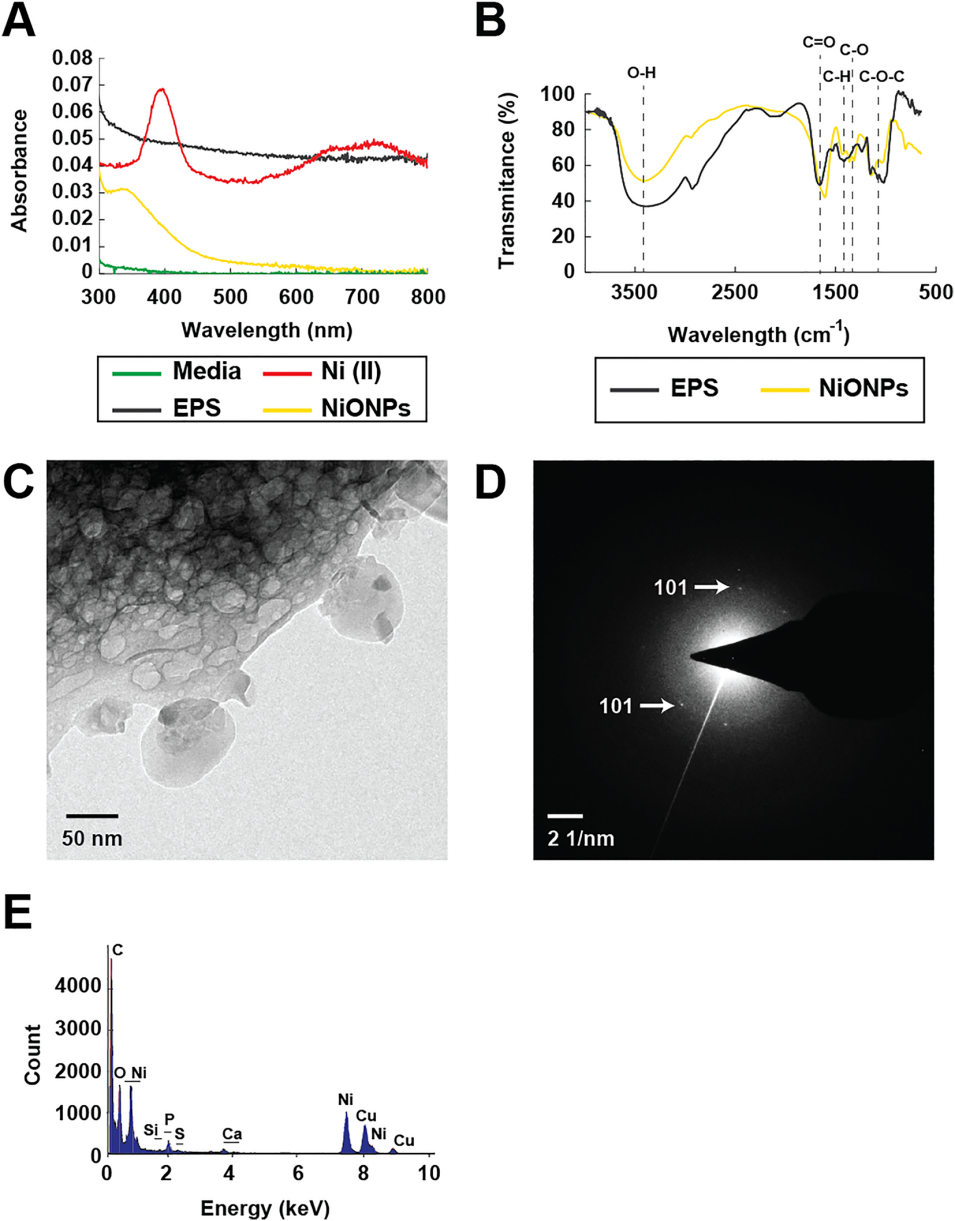
Characterization analysis of the synthesized NiO nanoparticles. (**A**) UV–Vis spectra. (**B**) FT-IR spectra. (**C**) TEM micrography of NiO nanoparticles in the EPS matrix. (**D**) SAED pattern of the synthesized NiO nanoparticles. (**E**) Elemental composition of the ESP-capped Ni nanoparticles.

NiONPs synthesis was made in an aqueous solution containing EPS from *R. mucilaginosa* as a capping agent and ascorbic acid as a reducing agent, adjusting the pH of the reaction media to 9 with NaOH. The synthesized nanoparticles were characterized using UV–Vis, FT-IR, TEM, SAED, and EDS (Fig. 1). UV–Vis shows the absorbance spectra of different reaction media containing the synthesized NiONPs as well as EPS alone, a Ni (II) solution, and a blank reaction made without a Ni(II) solution (Fig. 1A). A maximum absorbance peak of 348 nm is observed for NiONPs. Ni (II) solution has a strong peak of maximum absorbance at 395 nm, as well as two more peaks at 650–750 nm. When comparing the obtained spectra, the absorbance peaks of Ni (II) disappear in the NiONPs spectra, and a peak at 348 nm is present. Also, when comparing with EPS and blank reaction spectra, both having no absorbance peaks, it is notable that the obtained at 348 nm is caused by NiONPs presence and not due to possible modifications of the structure of EPS caused either by ascorbic acid or the pH modification.

Characterization using FT-IR shows few modifications in the EPS structure after the synthesis reaction, Fig. 1B. Both EPS and NiONPs show peaks at 3600–3200 cm^−1^ for O–H functional groups, C=O stretch at 1600 cm^−1^, 1450–1400 cm^−1^ for C–H bending in carbohydrate's backbones, C–O stretch at 1350–1300 cm^−1^, and the characteristic stretching vibration peaks at 1250–1050 cm^−1^ of sugar derivates. The NiONPs synthesis in the presence of EPS caused attenuation of peak intensities and wavenumber shifts of some EPS functional groups; the peak of C=O stretch is observed at lower wavelengths. Also, weaker C–H peaks are observed. In addition, the C–O–C peak, corresponding to sugar derivates, and the C–O peak become weaker and more potent.

TEM and SAED were also used to characterize the NiONPs. TEM images showed an average size of 26.73 nm for the synthesized nanoparticles over the EPS surface (Fig. 1C). SAED showed diffuse rings, as well as a ring with dots that allow the identification of a [1,0,1] plane (Fig. 1D). EDS analysis showed the element composition of the biocomposite, comprising Ni, C, and O as significant elements (Fig. 1E). EDS also shows Cu as the sample was analyzed over a copper grid and some Ca, P, and Si due to remaining debris of the containers where the NPs samples were stored.

For the antimicrobial assays, we first tested the antibiotics ampicillin, kanamycin, chloramphenicol, and ciprofloxacin for the inhibition assays against the clinical isolates to determine their individual MIC. Also, the MIC for these antibiotics against ATCC strains was assessed. The summary of the MICs obtained is displayed in Table 1. Then we tested the Ni-composite against the clinical isolates, as the ATCC were used to compare antibiotic susceptibility. The resulting Ni-composite MIC against the clinical isolates were 2 and 3 mg/mL against antibiotic-resistant *Pseudomonas aeruginosa* and *Staphylococcus aureus*, respectively. EPS alone did not affect the bacterial growth of the resistant bacteria at any concentration.

**Table 1:** The minimum inhibitory concentration of the different antibiotics.

Antibiotic	Minimum inhibitory concentration (ppm)					
	*PaR*	*Pa*	Fold increase	*SaR*	*Sa*	Fold increase
Ampicillin	8	16	–	512	0.0625	8192
Kanamycin	8192	16	512	8192	16	512
Chloramphenicol	128	8	16	64	1	64
Ciprofloxacin	32	0.125	256	32	0.125	256

*PaR* antibiotic resistant *Pseudomonas* *aeruginosa*, *SaR* antibiotic resistant *Staphylococcus* *aureus*, *Pa* *Pseudomonas* *aeruginosa*, *Sa* *Staphylococcus* *aureus.*

Combinations of 0, 0.125, 0.25, and 0.5 MIC of Ni-composite and antibiotics were tested, resulting in nine combination treatments, six individual treatments, and one untreated control. Each Ni-CACs was tested against multidrug-resistant *Staphylococcus aureus* and *Pseudomonas aeruginosa*.

**Figure 2:**
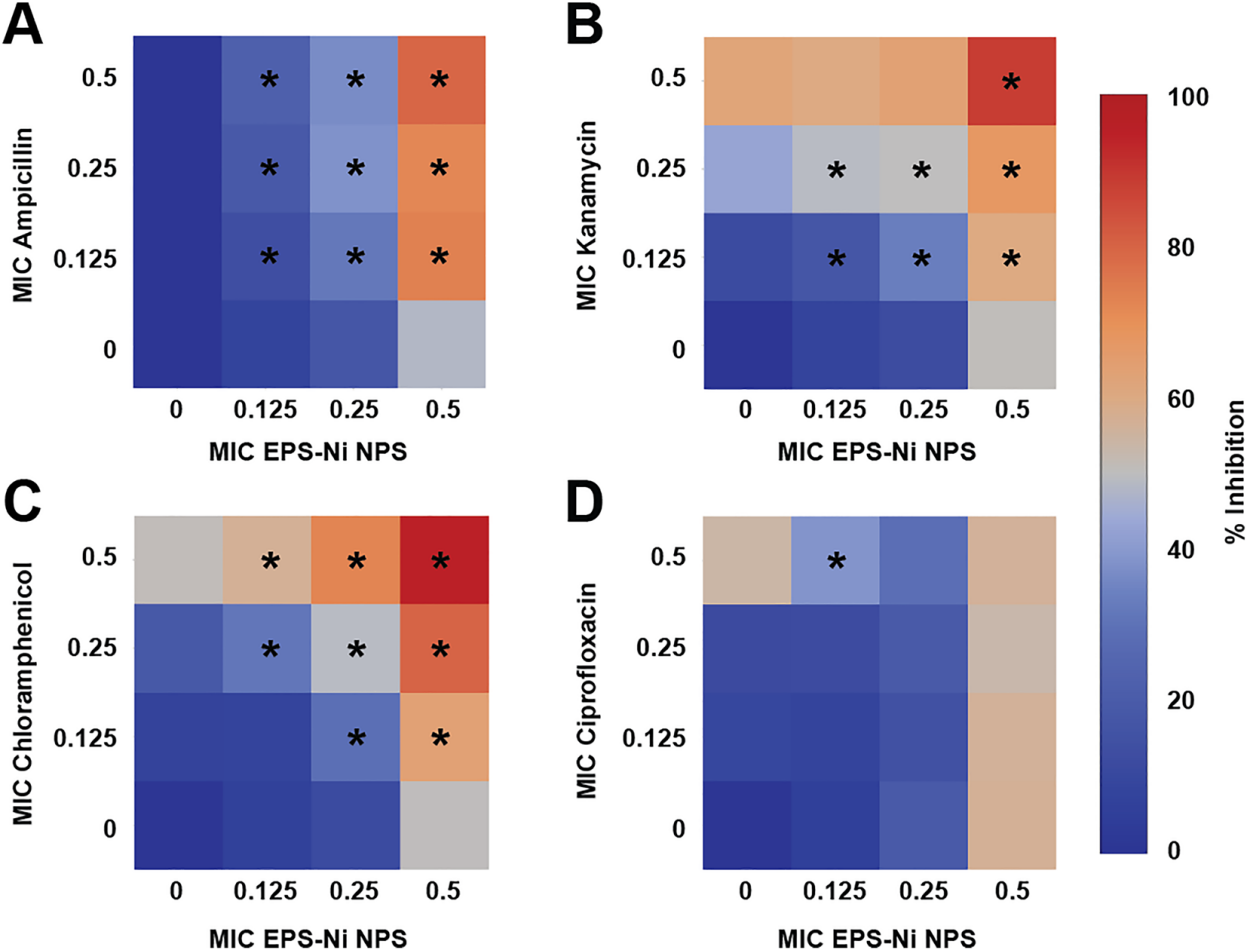
Inhibitory effect of the Ni-CACs using MIC fractions against multidrug-resistant *Staphylococcus aureus*. Inhibitory percentage caused by sub-inhibitory combinations of Ni-composite with (**A**) ampicillin, (**B**) kanamycin, (**C**) chloramphenicol, and (**D**) ciprofloxacin. (*) indicates a significant difference (p < 0.05) from individual treatment controls. Each checkerboard treatment, growth, and sterility control were performed in triplicates.

**Figure 3:**
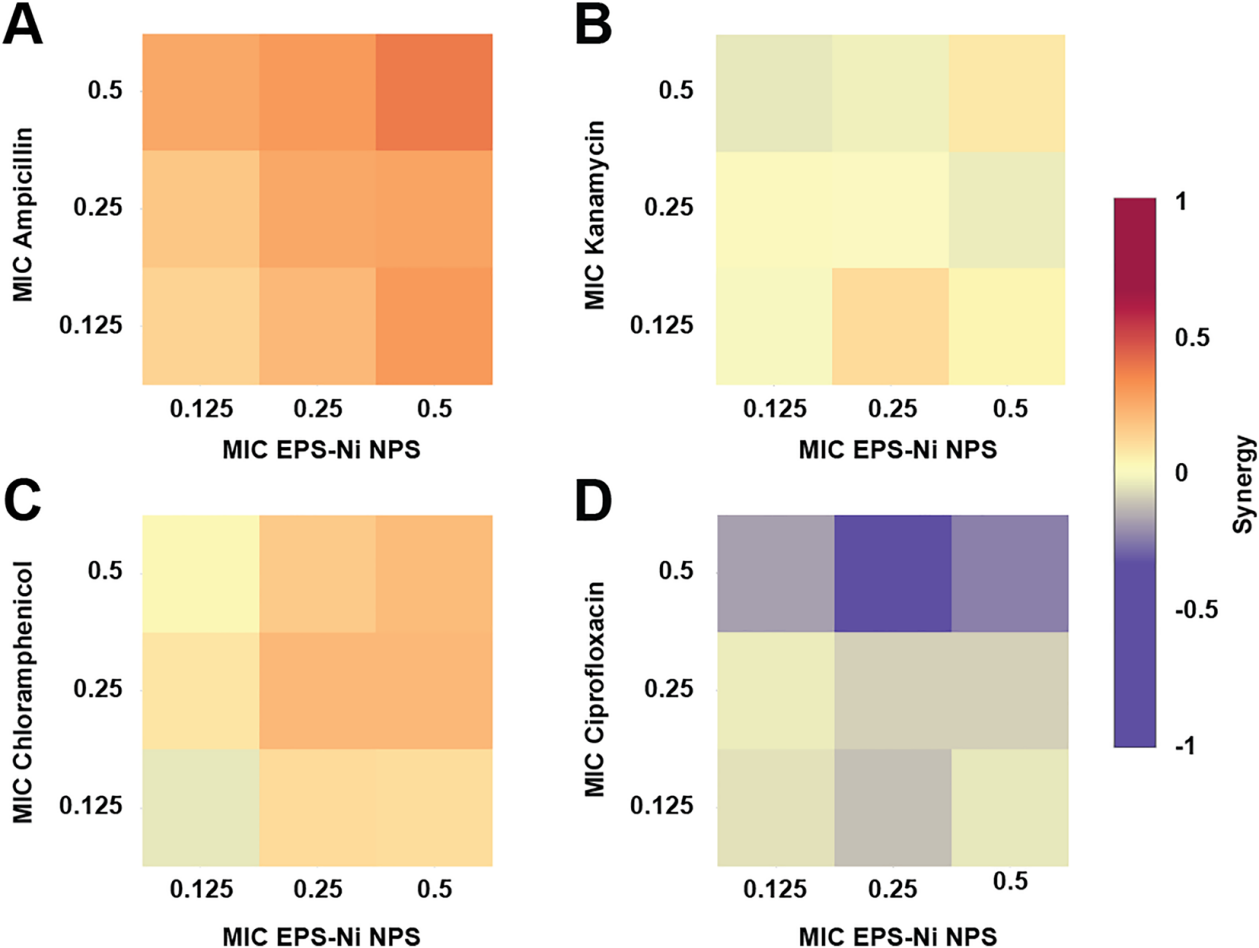
Analysis of Ni-CACs interactions against multidrug-resistant *Staphylococcus* *aureus*. Classification of the different interactions between Ni-composite with (**A**) ampicillin, (**B**) kanamycin, (**C**) chloramphenicol, and (**D**) ciprofloxacin. Interactions are classified as synergistic, additive, or antagonistic, with values > 0, = 0, and < 0, respectively. Each checkerboard treatment was done in triplicates.

Ni-composite combined with ampicillin, kanamycin, and chloramphenicol enhanced the antimicrobial effect against *SaR*. The synthesized Ni-composite enhanced significantly (p < 0.05) every combination made with ampicillin (Fig. 2A) from their respective control. More than 70% of the bacteria was inhibited when using 0.5 MIC of the composite with any of the MIC fractions of the antibiotic; around 31–36% of inhibition with the combinations using 0.25 MIC of the composite and 13–22% of inhibitions where 0.125 MIC of the composite was used. Most of the combinations of Ni-composite with kanamycin (Fig. 2B) showed significant differences (p < 0.05) compared with their controls. The combination of 0.5 MIC of both components exhibited almost 90% growth inhibition, while 0.5 MIC of the Ni-composite with 0.125 and 0.25 MIC of kanamycin inhibited 60 and 67%, respectively. Combining 0.25 MIC of Ni-composite with 0.125 and 0.25 MIC of kanamycin showed growth inhibition of 33% and 49%, respectively. Last, 0.125 MIC of Ni-composite with 0.125 and 0.25 MIC of kanamycin inhibited 17 and 48%, respectively. On the other hand, the combination of Ni-composite with chloramphenicol (Fig. 2C) showed significant differences (p < 0.05). Growth inhibition of 95% was observed when *SaR* was treated with 0.5 MIC of each component, while 80% growth inhibition showed at 0.5 and 0.25 MIC of the Ni-composite and chloramphenicol, respectively. The combination of Ni-composite with ciprofloxacin (Fig. 2D) only showed a growth inhibition when 0.125 MIC of the Ni-composite was combined with 0.5 MIC of ciprofloxacin (p < 0.05). Interestingly, most of the combinations of Ni-composite with ampicillin, kanamycin, and chloramphenicol (Fig. 3A–C) presented a synergistic effect (S value > 0). Moreover, the synergistic effect observed in the combination of Ni-composite with ampicillin and chloramphenicol was in a dose–response manner; meanwhile, the combination with ciprofloxacin showed mostly antagonistic effects (Fig. 3D) (S value < 0).

**Figure 4:**
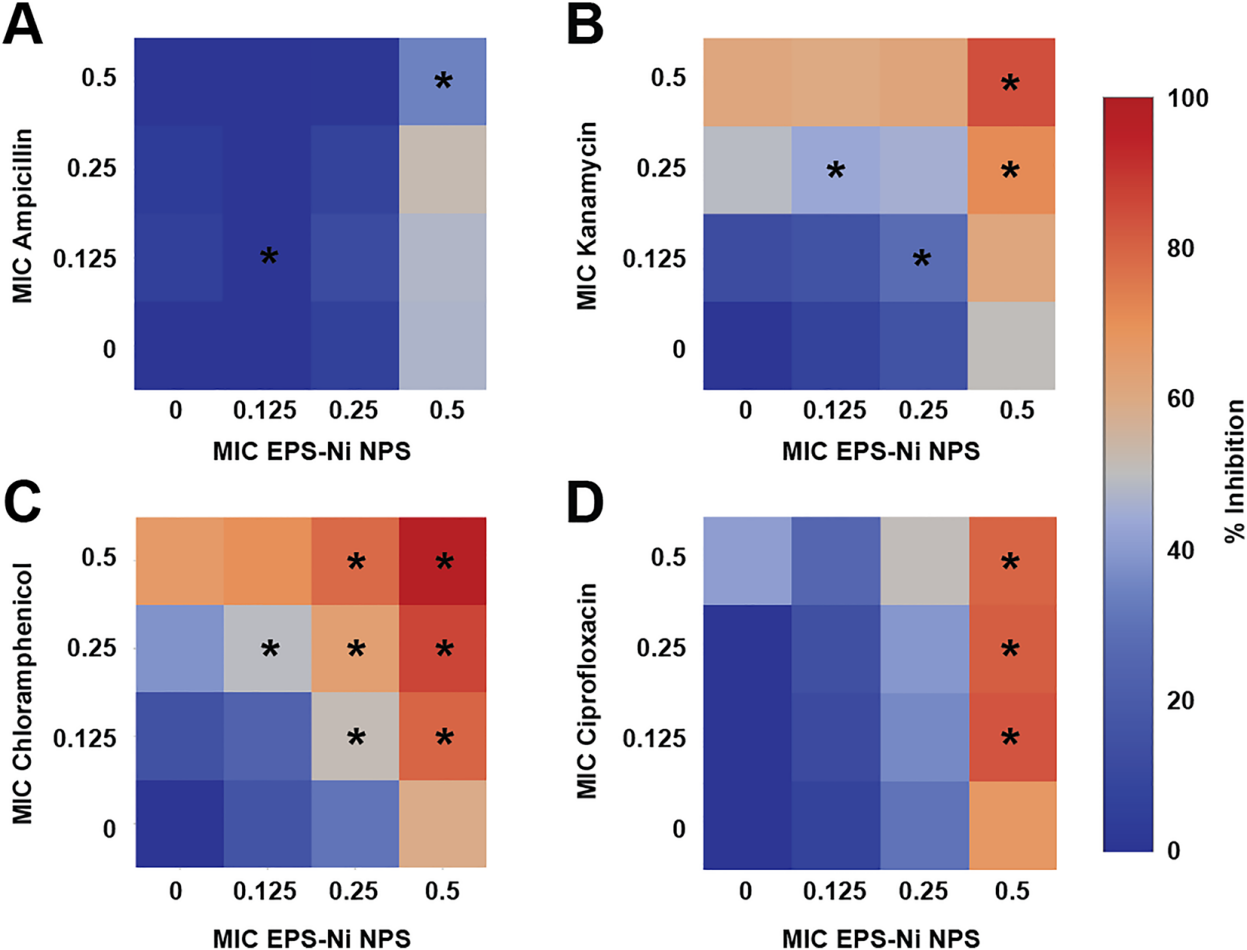
Inhibitory effect of the Ni-CAC using MIC fractions against multidrug-resistant Pseudomonas aeruginosa. Inhibitory percentage caused by sub-inhibitory combinations of Ni-composite with (**A**) ampicillin, (**B**) kanamycin, (**C**) Chloramphenicol, and (**D**) ciprofloxacin. (*) indicates a significant difference (p < 0.05) from individual treatment controls. Each checkerboard treatment, growth, and sterility control were performed in triplicates.

In addition, we evaluated the antibacterial effect of Ni-composite combined with antibiotics (ampicillin, kanamycin, chloramphenicol, and ciprofloxacin) against multidrug-resistant *Pseudomonas aeruginosa* (a Gram-negative bacterium) (Fig. 4 A-D). The combination of Ni-composite with kanamycin, chloramphenicol, and ciprofloxacin enhanced antibacterial activity against *PaR*. When Ni-composite was combined with kanamycin (Fig. 4B), four out of nine combinations showed significant differences (p < 0.05) from their controls. 0.5 MIC of Ni-composite combined with 0.25 and 0.5 MIC of kanamycin increased the growth inhibition by 71% and 84%, respectively. Also, the two remaining combinations resulted in almost 50% of growth inhibition. Then, most of the combinations of Ni-composite/chloramphenicol (Fig. 4C) exhibited a significant growth inhibition (p < 0.05) compared with their controls. The fractions 0.25 and 0.5 MIC of Ni-composite combined with every MIC fraction (0.125, 0.25, and 0.5) of chloramphenicol increased the growth inhibition of *PaR*. For example, 0.5 MIC of Ni-composite combined with 0.125, 0.25, and 0.5 MIC of chloramphenicol showed an inhibitory effect of 80, 86, and 97%, respectively. While Ni-composite combined with ciprofloxacin (Fig. 4D) only showed significant differences (p < 0.05) in three combinations. The fraction 0.5 MIC of Ni-composite combined with 0.125, 0.25, and 0.5 MIC of ciprofloxacin showed 83, 81, and 80% of bacterial growth inhibition, respectively. Last, the combination of Ni-composite/ampicillin (Fig. 2A) only presented a significant difference (p < 0.05) in the two groups; however, this difference did not show a relevant antibacterial effect.

**Figure 5:**
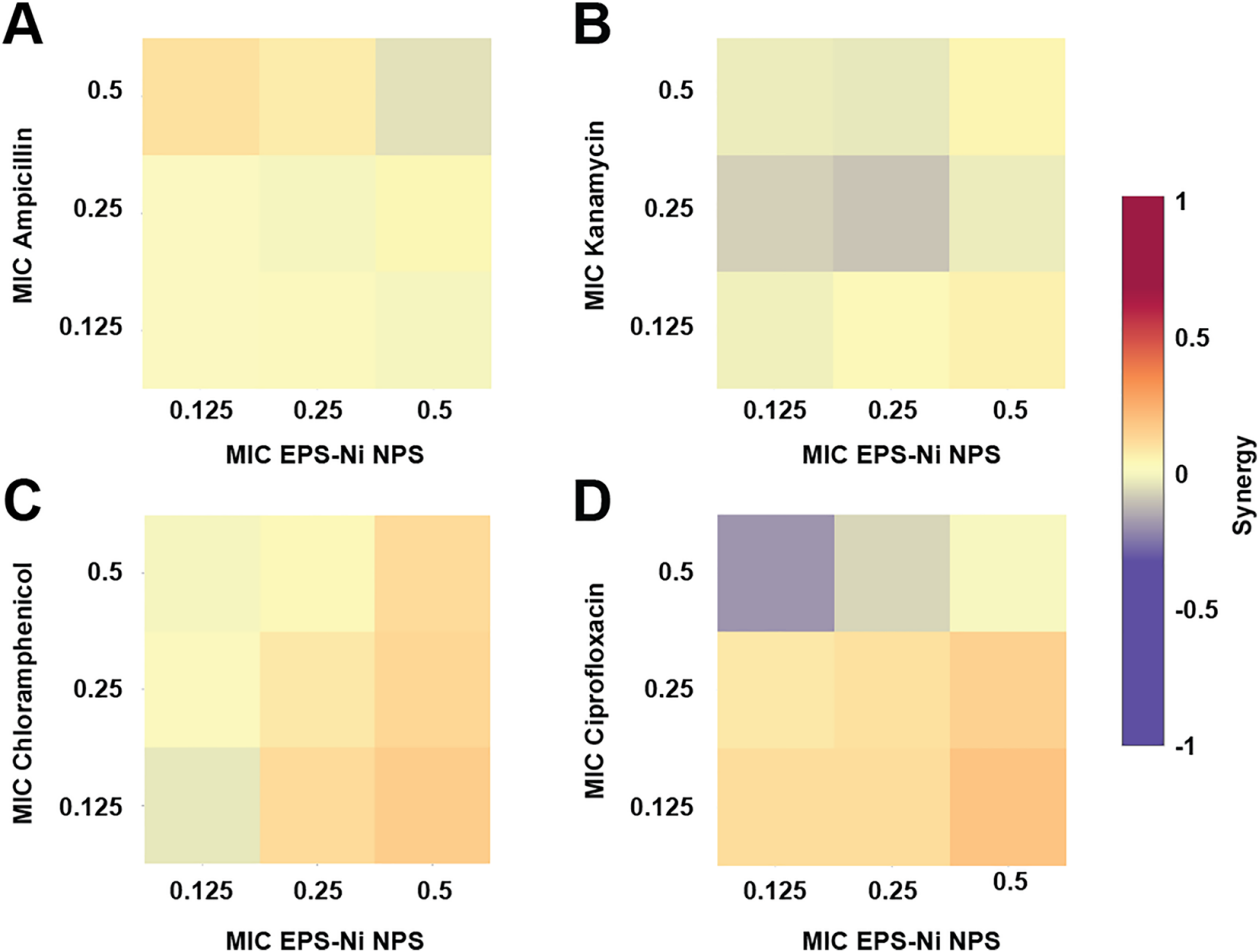
Analysis of Ni-CACs interactions against multidrug-resistant *Pseudomonas* *aeruginosa*. Classification of the different interactions between Ni-composite with (**A**) ampicillin, (**B**) kanamycin, (**C**) chloramphenicol, and (**D**) ciprofloxacin. Interactions are classified as synergistic, additive, or antagonistic, with values > 0, = 0 and < 0, respectively. Each checkerboard treatment was done in triplicates.

The results demonstrate interesting synergistic and antagonistic drug interactions between these compounds. For example, the combination of Ni-composite with ampicillin (Fig. 5A) exhibited an antagonistic interaction. The antibacterial effect decreased when the MIC fractions of Ni-composite increased, and the fraction of ampicillin was 0.5 MIC. The combinations with kanamycin and chloramphenicol (Figs. 5B,C) showed a synergistic effect in a concentration-dependent manner when Ni-composite concentration was increased. Nonetheless, a double trend was observed in combination with ciprofloxacin (Fig. 5D). When Ni-composite concentration was increased, the interaction became synergistic; while increasing antibiotic concentration, it turned antagonistic.

**Figure 6:**
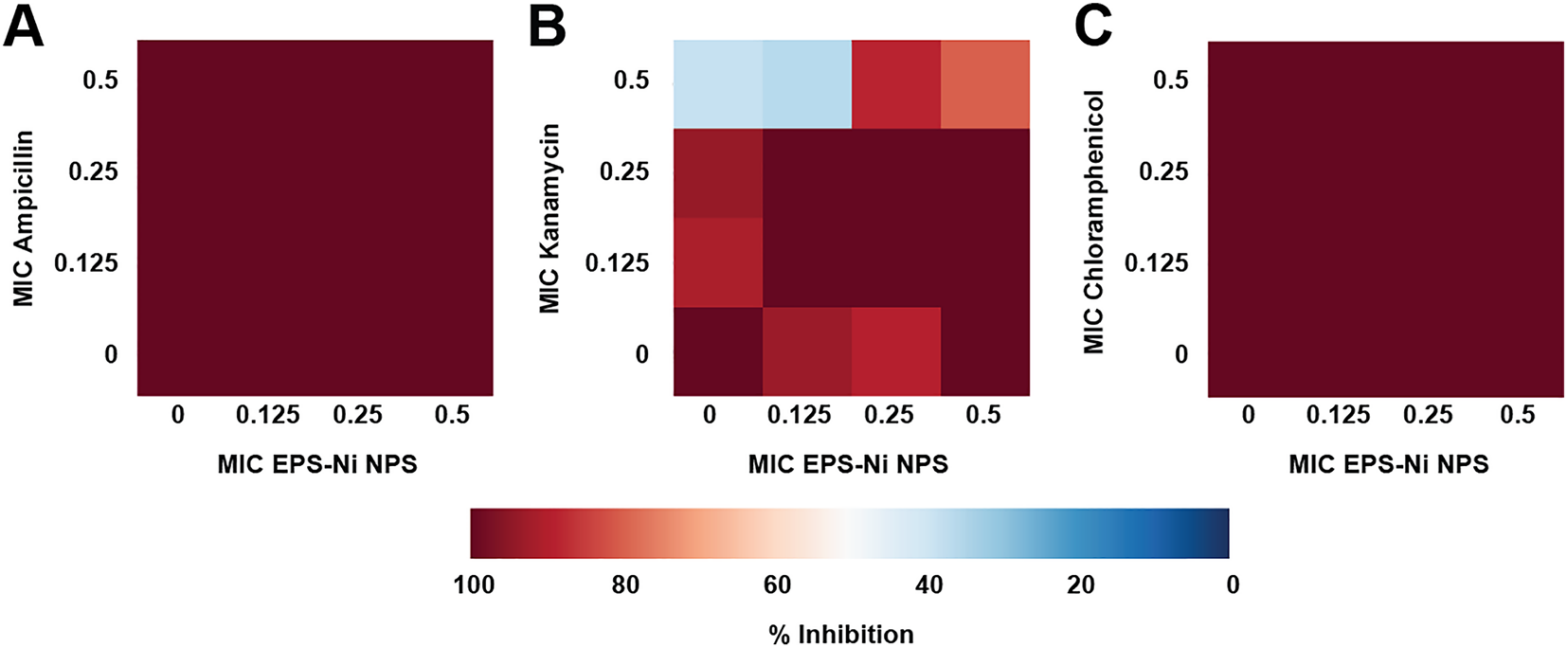
Effect on biofilm production caused by Ni-CACs against multidrug-resistant *Staphylococcus* *aureus*. Biofilm production percentage caused by sub-inhibitory combinations of Ni-composite with (**A**) ampicillin, (**B**) kanamycin, and (**C**) Chloramphenicol. Each checkerboard treatment, growth, and sterility control were performed in triplicates.

**Figure 7:**
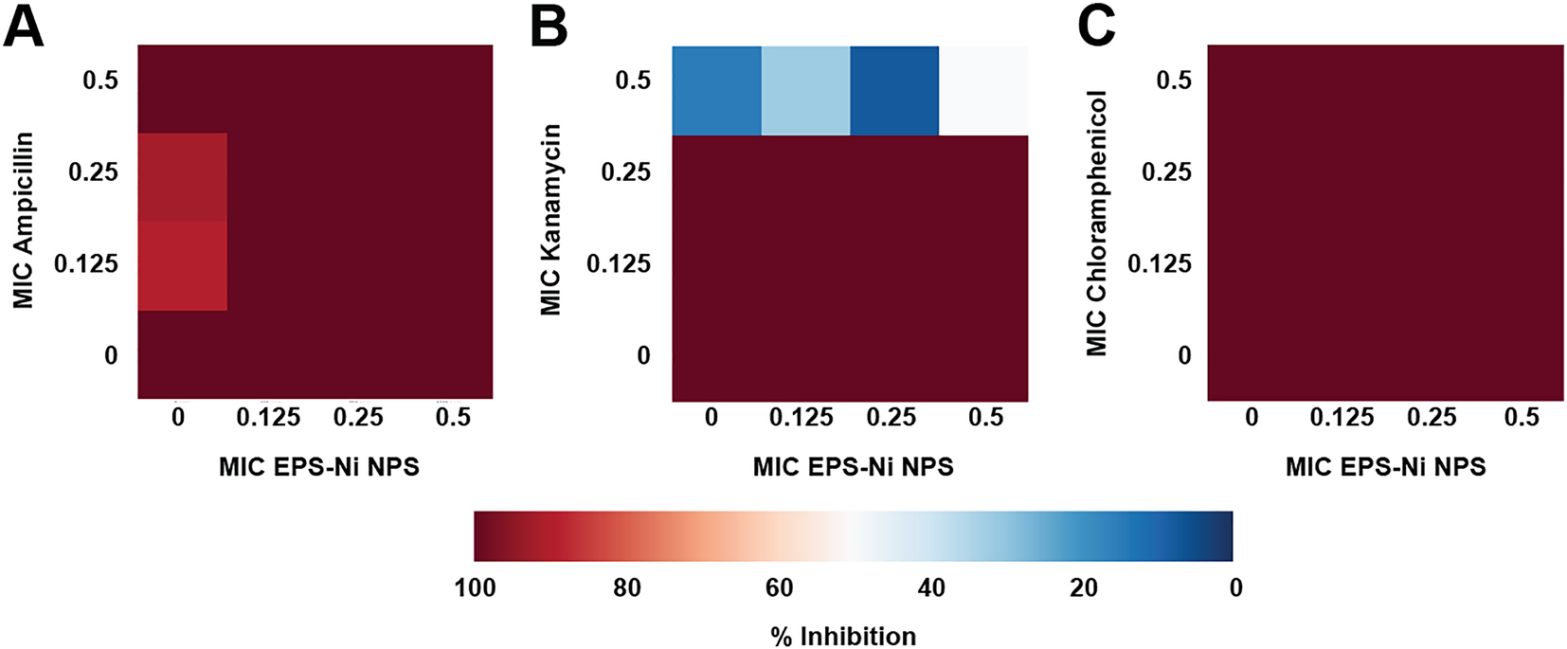
Effect on biofilm production caused by Ni-CACs against multidrug-resistant *Pseudomonas aeruginosa*. Biofilm production percentage caused by sub-inhibitory combinations of Ni-composite with (**A**) ampicillin, (**B**) kanamycin, and (**C**) Chloramphenicol. Each checkerboard treatment, growth, and sterility control were performed in triplicates.

Also, we evaluate the capacity of Ni-composite combined with ampicillin, kanamycin, and chloramphenicol to inhibit the biofilm production of *Staphylococcus aureus* and *Pseudomonas aeruginosa* strains. Most of the combinations did not affect the biofilm production of both strains (Figs. 6 and 7). Combining kanamycin with specific MIC fractions of Ni-composite (0.125 and 0.25) resulted in an inhibition of biofilm production, registering approximately 60–80% inhibition in *S. aureus* and *P. aeruginosa*, respectively. However, this exhibited no significant difference when compared to their corresponding controls. Notably, throughout the entire duration of the antibiofilm experiments, there was an absence of precipitate formation within the test wells. It's worth mentioning that the anticipated enhanced effect of ciprofloxacin was distinctly observed solely against Gram-negative bacteria. Consequently, this particular antibiotic was not employed in combination with the Ni-composite for the antibiofilm tests.

## Discussion

To ensure that we have synthesized NiO nanoparticles we use different characterization techniques. UV–Vis spectra reveals characteristic light absorption of different materials, like metallic nanoparticles, due to the surface plasmon resonance as an interactions of the electrons in the metallic nanoparticle surface with incident photons. We observed maximum absorbance peaks at 395 nm, as well as two more peaks at 650–750 nm, proper of Ni (II) solution^23^. When comparing the obtained spectra with the obtained in the synthesis reaction, the absorbance peaks of Ni (II) disappear in the NiONPs spectra, and a peak at 348 nm is present, characteristic of NiO nanoparticles^24–26^. Characterization using FT-IR shows few modifications in the EPS structure after the synthesis reaction. Both EPS and NiONPs show characteristic peaks proper of microbial exopolysaccharides. Both compounds showed characteristic stretching vibration peaks at 1250–1050 cm^-1^ of sugar derivates suggesting that the EPS mainly comprises saccharides and carboxylates, as reported previously^27^. The NiONPs synthesis in the presence of EPS caused attenuation of peak intensities and wavenumber shifts of some EPS functional groups caused by the redox reaction. Due to the conjugation of the synthesized nanoparticles and EPS matrix, the peak of C=O stretch is observed at lower wavelengths. Also, weaker C–H peaks are observed due to metallic nanoparticles' interaction with the polysaccharide's hydrogen bonds^28^. In addition, the C–O–C peak, corresponding to sugar derivates, and the C–O peak become weaker and more potent. These changes suggest that some of the sugars that form the EPS and the ascorbic acid are participating in the redox reaction needed in synthesizing NiONPs^29^. The SAED showed diffuse rings due to the amorphous composition of EPS, as well as a ring with dots that allow the identification of a [1,0,1], characteristic of NiO nanoparticles with rhombohedral geometry (JCPDS No. 44-1159)^30,31^.

In the preceding discussions, it has been elucidated that the antibacterial efficacy of nickel nanoparticles has undergone rigorous testing across a diverse array of microorganisms, predominantly ATCC strains such as *B. subtilis*, *B. licheniformis*, *E. coli*, and *K. pneumoniae*^15–17,14^. However, it is imperative to accentuate that clinical isolates often demonstrate superior clinical relevance in contrast to ATCC strains. This distinction arises because the former are typically susceptible to available antibiotics^32,33^, whereas the latter, especially antibiotic-resistant strains, pose the potential threat of instigating human infections, subsequently leading to adverse health consequences^34^. Combining antimicrobial compounds based on clinically proven recommendations and scientific research is a promising and effective way to overcome multidrug resistance, as it can restore the susceptibility of different bacteria to antibiotics. Using two or more antimicrobial compounds could lead to a synergistic effect and enhance treatment effectivity^35–40^. In our endeavor to elucidate the intricacies of this phenomenon, we have employed the Bliss independence criterion as a stringent evaluative tool to assess the interplay among the antimicrobial entities under study. This analytical model is instrumental not only in quantifying their interactions but also helps to discern how these compounds potentially act when functioning independently. It is pertinent to highlight that our hypothesis lies in the distinct antimicrobial pathways employed by antibiotics and metal nanoparticles, leading to the synergistic phenomenon observed. Many compounds have been introduced as adjuvants to enhance the antibacterial activity of existing antibiotics^41^. Silver nanoparticles (AgNPs) have been reported to have the potential to enhance the antimicrobial activity of antibiotics^42^. For example, combining Beta-Lactam antibiotics or aminoglycosides with AgNPs enhanced bactericidal activity against Gram-positive and Gram-negative bacteria^43^. Another study observed that combinations of AgNPs or CuNPs (copper nanoparticles) with either tetracycline or kanamycin could increase or decrease the antimicrobial activity against *Bacillus subtilis* and *Pseudomonas flourescens*^44^. We have previously reported that combining AgNP and ZnONP (zinc oxide nanoparticles) with antibiotics showed different effects depending on the type of bacteria. For example, AgNPs combined with ampicillin showed different inhibition effects between Gram-positive and Gram-negative bacteria. Moreover, ZnONPs combinations with ampicillin showed antagonistic effects against the tested bacteria^45^.

In the present study, this behavior was also observed when combining Ni-composite with antibiotics since the antibacterial activity was modified. The differences may be related to the bacteria's particular sensitivity to antibiotics and the interaction with the Ni-composite. The higher sensibility of Gram-positive bacteria to Beta-Lactam antibiotics may lead to the synergistic effects observed in our study. *SaR* was highly inhibited by Ni-composite combined with ampicillin, unlike *PaR*, where antagonistic effects were observed. As reported in previous work^11^, the Gram-negative strain was more sensitive to the Ni-composite, needing a lower concentration to achieve a complete growth inhibition, reducing the possibility of forming complexes with the antibiotics.

Regarding the combinations with ciprofloxacin, we observed a different behavior from our initial expectations. Ciprofloxacin, characterized as a broad-spectrum antibiotic, typically exerts growth inhibitory effects on both Gram-positive and Gram-negative bacterial strains. Notably, only a few combinations of Ni-composite with ciprofloxacin exhibited a significant dose-dependent response. All combinations showed synergistic effects at low antibiotic concentrations, but when antibiotic concentration was increased, the interaction was antagonistic with every combination of 0.5 MIC of antibiotic.

The exact mechanism of the antimicrobial effect of NiONPs remains uncertain. Studies indicate it could be associated with releasing Ni^2+^ ions and ROS activity^46^. Other studies have reported that the antimicrobial effect is mainly caused by the interaction of the NiONPs with bacteria membranes and not by released ions^11,47^. In addition, green synthesized NiONPs can interact with cell membranes causing alterations in cell surface morphology^11,47^. Also, the Ni-composite induces antimicrobial activity by compromising cell membrane integrity, causing leakage of intracellular compounds (proteins, nucleic acids, K^+^, Mg^2+^)^11^. Despite the fact that clinically isolated bacteria may possess more than one mechanism of antibiotic resistance, that most common intrinsic resistance mechanisms encompass enhanced activity of efflux pumps and a reduced permeability of the outer membrane^48,49^. Thus, we hypothesize that the enhanced effect of the antibiotics can be explained in terms of cell membrane integrity. Ni-composite could cause alterations in the outer layer (membrane) of antibiotic-resistant bacteria, and soluble extracellular molecules can cross the membrane into the intracellular space more easily than when membrane destabilization does not occur. For this reason, we propose that Ni-composite allows more antibiotic entrance to the bacterial cell, resulting in a higher antimicrobial effect than without the presence of the composite, in addition to the possible leakage of vital intracellular compounds.

Within the realm of antibiotic research, it has been discerned that certain antibiotics are capable of forming complexes with metal ions. These formed complexes can either augment or attenuate the antimicrobial efficacy of the antibiotic in question^50,51^. Notably, interactions between ciprofloxacin and metals have been documented to form distinct complexes, manifesting either as ions^52–54^ or nanoparticles^52–55^ which subsequently influence their antimicrobial dynamics. In the context of our experimental design, it is interesting that the Ni-composite, in combination with ciprofloxacin, underwent complexation either intracellularly or extracellularly. This potential interaction might have inadvertently compromised their antimicrobial activity, thus elucidating the observed antagonistic interactions with diminished antimicrobial activity when administered in combination.

Commonly biofilm production is induced by external stress, like high temperature and osmolarity, and this includes the presence of subinhibitory concentrations of antimicrobial agents. Here, we undertook the task of evaluating the antimicrobial and antibiofilm activity of subinhibitory concentrations of a variety of antibiotics in combination with a Ni-composite. Contrary to what we expected, while the antimicrobial activity was manifestly present, an antibiofilm effect remained elusive. A parallel phenomenon was delineated by Lagha et al.^56^, during their investigation of various essential oils as potential antimicrobial and antibiofilm agents. Their findings revealed pronounced antimicrobial properties across different essential oil concentrations, yet a conspicuous absence of antibiofilm activity at analogous concentrations. Conversely, research by Soliman et al.^57^ identified antibiofilm activity at concentrations where antimicrobial effects were undetected. Furthermore, certain instances have been reported where subinhibitory antibiotic concentrations inadvertently bolstered biofilm formation in different *Staphylococcus* and *Pseudomonas* strains^58–60^. Such behaviors might offer insight into our findings wherein subinhibitory combinations of Ni-CACs resulted in nominal, if any, decrement in biofilm production. This is observed in its synergy with kanamycin, which, when juxtaposed with untreated controls for both strains, demonstrated antimicrobial action but lacked antibiofilm effects. Although the effect on biofilm production was not as expected, the main objective of this study was centered on enhancing the antimicrobial potential of antibiotics, particularly in the milieu of organisms that have already developed resistance. Our main goal is not to prevent antibiotic resistance in the clinical setting but rather to open the landscape to a promising avenue, which is the re-emergence of pre-existing antibiotics, positioning them as viable alternatives in our search for new antimicrobial agents.

The results obtained, particularly the observed improvement in the efficacy of several antibiotics against resistant strains, underscores the significant potential of our research in contributing to the scientific community's understanding of antimicrobial resistance. The intricate effects of synthetically derived compounds, such as those examined in our study, when used as adjuncts to established antibiotics, underscore the complexity and promise of this approach in addressing the escalating challenge of antimicrobial resistance.

The use of these kinds of methodologies, like checkerboard assays, could guide researchers to find a way to overcome antibiotic resistance. As seen above, some combinatorial treatments could lead to synergistic interactions giving the possibility of finding new strategies to fight superbugs. Nevertheless, it is also notable that not all combinatorial treatments can sensitize resistant bacteria to an antibiotic. Some combinations, even showing promising effects at certain combinations, could lead to an antagonistic effect, decreasing the significant effect of the antimicrobial compounds.

## Conclusion

A Ni-composite, synthesized using *Rhodotorula mucilaginosa* UANL-001L exopolysaccharide as a capping agent to NiO nanoparticles, was used to enhance the antimicrobial effect of different antibiotics against two multidrug-resistant strains, a Gram-positive and a Gram-negative bacterium. Ni-composite enhanced the antimicrobial effect of kanamycin and chloramphenicol against multidrug-resistant *Staphylococcus aureus* and *Pseudomonas aeruginosa*, reducing the antibiotic concentration needed by a quarter. Ni-composite combined treatment reduced the ampicillin concentration required to inhibit multidrug-resistant *Staphylococcus aureus* by eight. The combination of Ni-composite with ciprofloxacin showed a reduction of concentration (one-eighth) of antibiotic required to inhibit multidrug-resistant *Pseudomonas aeruginosa*. Interestingly, most of the combinatorial treatments showed a synergistic effect. Ni-composite could not enhance the antimicrobial effect of ciprofloxacin and ampicillin against multidrug-resistant *Staphylococcus aureus* and *Pseudomonas aeruginosa*, respectively. Similarly, Ni-CACs could not reduce biofilm production when tested against both resistant strains.

Although our study sheds light on the promising synergistic results of combining nickel nanoparticles and various antibiotics, it refrains from probing the specific resistance mechanisms attributable to each antibiotic. However, this opens a fertile path for future research aimed at deciphering the impact of distinct resistance mechanisms on the dynamics of synergy or antagonism in such combinations. The enhanced antimicrobial activity observed in this study could be attributed to the capacity of Ni-composite to destabilize the cell membrane, giving more accessible access to the antibiotic to enter the bacterial cell. Our results conclude that this Ni-composite can be used as an enhancer of the antimicrobial activity of various antibiotics. Nevertheless, further studies are needed to assess the innocuity of these Ni-CACs in vivo, focused on infections caused by multidrug-resistant strains.

## Data Availability

All data generated or analyzed during this study are included in this published article.
